# HandiVIH—A population-based survey to understand the vulnerability of people with disabilities to HIV and other sexual and reproductive health problems in Cameroon: protocol and methodological considerations

**DOI:** 10.1136/bmjopen-2015-008934

**Published:** 2016-02-04

**Authors:** Pierre De Beaudrap, Estelle Pasquier, Alice Tchoumkeu, Adonis Touko, Frida Essomba, Aude Brus, Annabel Desgrées du Loû, Toyin Janet Aderemi, Jill Hanass-Hancock, Arne Henning Eide, Daniel Mont, Muriel Mac-Seing, Gervais Beninguisse

**Affiliations:** 1 IRD, CEPED, UMR 196, Université Paris Descartes—Institut de Recherche pour le Développement (IRD), Paris, France; 2 Initiative 5% Sida, Tuberculose, Paludisme/Expertise France, Paris, France; 3 Institut de Formation et de Recherche Démographique (IFORD), Yaoundé, Cameroon; 4 Forum Camerounais de Psychologie, Yaoundé, Cameroon; 5 Handicap International, Lyon, France; 6 Ayahulet Consulting, Abuja, Nigeria; 7 HEARD, Pretoria, South Africa; 8 SINTEF Technology and Society, Oslo, Norway; 9 Leonard Cheshire Disability and Inclusive Development Centre, University College London, London, UK

**Keywords:** EPIDEMIOLOGY, STATISTICS & RESEARCH METHODS

## Abstract

**Introduction:**

In resource-limited countries, people with disabilities seem to be particularly vulnerable to HIV infection due to barriers to accessing information and services, frequent exposure to sexual violence and social exclusion. However, they have often been left behind in the HIV response, probably because of the lack of reliable epidemiological data measuring this vulnerability. Multiple challenges in conducting good quality epidemiological surveys on people with disabilities require innovative methods to better understand the link between disability and HIV. This paper describes how the design and methods of the HandiVIH study were adapted to document the vulnerability of people with disabilities to HIV, and to compare their situation with that of people without disabilities.

**Methods and analysis:**

The HandiVIH project aims to combine quantitative and qualitative data. The quantitative component is a cross-sectional survey with a control group conducted in Yaoundé (Cameroon). A two-phase random sampling is used (1) to screen people with disabilities from the general population using the Washington Group questionnaire and, (2) to create a matched control group. An HIV test is proposed to each study participant. Additionally, a questionnaire including a life-event interview is used to collect data on respondents’ life-course history of social isolation, employment, sexual partnership, HIV risk factors and fertility. Before the cross-sectional survey, a qualitative exploratory study was implemented to identify challenges in conducting the survey and possible solutions. Information on people with disabilities begging in the streets and members of disabled people's organisations is collected separately.

**Ethics and dissemination:**

This study has been approved by the two ethical committees. Special attention has been paid on how to adapt the consenting process to persons with intellectual disabilities. The methodological considerations discussed in this paper may contribute to the development of good practices for conducting quantitative health surveys on people with disabilities.

**Trial registration number:**

NCT02192658.

**Strengths and limitations of this study**

- Limitations include the difficulties to identify people with disabilities due to the limitation of the screening tool and the absence of a single and objective definition of disability.
- Strengths include the complex random sampling method to prevent selection bias;
- This study presents the use of a life-event history approach to understand the life trajectories of people with disabilities and how it relates to their vulnerability to HIV and the use of innovative communication methods to conduct the interviews that will improve the quality of data collected and decrease the risk of bias.

## Background

As highlighted during the 2014 International AIDS Conference held in Melbourne, there will be no ending of the HIV epidemic without closing the gap between people included in the global AIDS response and those left behind.^1^ In many resource-limited countries, people with disabilities are believed to be highly vulnerable to HIV infection;^2^ ^3^ many face multiple barriers to access HIV and sexual and reproductive health (SRH) information.^4–9^ A growing body of evidence shows that people with disabilities are more likely to be victims of violence and abuse, which have been shown to be significantly associated with an increased risk of HIV infection.^2^ ^10–13^ Disadvantages such as a lack of education and resources as well as social exclusion that are often experienced by people with disabilities in resource-limited countries have a negative impact on their ability to maintain good health. These factors and others contribute to creating a global risk environment.^14–16^ Although this vulnerability was first recognised 10 years ago and stressed in several international statements,^17–22^ people with disabilities have often been left behind with regard to the prevention and treatment of HIV/AIDS.^1^ ^13^ ^23^ A potential explanation for that is the lack of reliable epidemiological data measuring this vulnerability. Results from various reviews including a recent meta-analysis of studies conducted in Sub-Saharan Africa suggest that adults with disabilities have at least the same risk of HIV infection compared to the general population in this part of the world.^2^ ^3^ ^24^ ^25^ However, given the limited quality of available studies and the variety of methodologies used,^24^ more epidemiological research is needed to establish firm evidence and to better understand the complex links between disability and HIV in order to help decision-makers prioritise their interventions. This paper presents the HandiVIH study design, as well as the methodological challenges of research aiming at providing such quantitative evidence.

### Multiple definitions of disability and implications for research

According to the World Report on Disability, people with disabilities represent around 15% of the world's population, with 80% living in resource-limited countries.^13^ However, large variations in disability prevalence are observed between studies, which could be explained by the different definitions of disability used.^26^ Disability is complex, dynamic and multidimensional.^13^ ^27^ ^28^ As a result, instead of a single, objective and easy-to-measure definition of disability, there are multiple approaches that only partially overlap.^29^ ^30^ The medical approach that prevailed for decades focused only on impairments and their causes. This approach has been challenged by people with disabilities and several academic writers.^31^ A conceptual shift was operated through the social model in which people are viewed as being disabled mainly because of environmental barriers that prevent full participation in society.^32^ Integrating all these thoughts, the so-called ‘bio-psycho-social’ model promoted by the WHO proposes that disability is constructed of three connected components (impairments, activity limitations and social participation restrictions), and results from the interaction between individuals and environmental factors.^13^ ^33^ This model requires different levels of information (individual, micro and macro environment) to capture the disability experience, and necessitates that researchers use and combine different methods and approaches.

### People with disabilities—a hard-to-survey population

People with disabilities living in resource-limited settings can be considered as a hard-to-survey population because of the technical challenges in identifying them,^34^ difficulty in establishing a sampling frame, and the existence of widespread exclusion mechanisms. Most studies available on HIV try to overcome such difficulties by using non-probabilistic sampling methods such as ‘snowball’ sampling.^35^ However, these methods do not allow for valid statistical inferences and lead to selection biases. One available alternative is a two-phase random sampling design with people with disabilities screened from the general population during the first phase and their eligibility confirmed during the second phase.^36^ ^37^ However, particular attention needs to be paid to the screening questionnaire that should have acceptable sensitivity, specificity, validity and accuracy.^36^ ^38^ This could be challenging in the context of disability. First, the wording of questions can create stigma and, as a result, people may be reluctant to identify themselves as disabled, or household members may experience shame at having a member with a disability and thus avoid identification.^39^ Second, the questions themselves may identify some disability types better than others. Third, the research communication process itself may be inaccessible for some groups of people with disabilities such as those with intellectual disabilities or the deaf-blind.

### Methods to investigate the vulnerability of people with disabilities

The vulnerability of people with disabilities to HIV and SRH adverse outcomes might be multifactorial, involving individuals as well as contextual factors that occur at different times during their life course. Therefore, to understand the link between disability and HIV infection, it is necessary to move beyond a static cross-sectional analysis and adopt a life-course approach. Such an approach aims to collect information on events and experiences over the lifetime, in order to identify sequences of risks and patterns of life-course trajectories, and to examine their association with health outcomes.^40^ ^41^ Although longitudinal studies are the gold standard approach for life-course research, they are difficult to implement in low-income contexts, particularly because of high sample attrition and relatively high implementation costs. Alternatively, retrospective life-course studies have been shown to be useful and to provide data with adequate accuracy.^42–44^

### Objectives and assumption of the HandiVIH study

This study aims at improving our understanding of the situation of people with disabilities in Sub-Saharan Africa in relation to HIV and their SRH. The primary objectives of this study are to compare quantitatively the risk of HIV infection among people with disabilities to those without and to analyse the factors associated with their vulnerability to HIV. To do that, the study will (1) compare the HIV prevalence among people with disabilities and matched controls, (2) explore in detail their life-course events and knowledge, attitudes and practices, as well as (3) their access to information/services in relation to HIV and SRH, including their ability to negotiate safer sex and their exposure to violence. As the social environment is thought to be an important determinant of the vulnerability to HIV,^45^ ^46^ the study will examine some aspects of people with disabilities' social environment, namely their social network and social participation.

Additional information on people with disabilities will be obtained through two substudies assessing people with disabilities begging in the streets and those who are members of disabled people's organizations (DPO). They aim to explore the boundary of the main study population and to assess the vulnerability to HIV of people with disabilities from these two groups.

## Methods and design

### Study strategy and design

**Figure 1:**
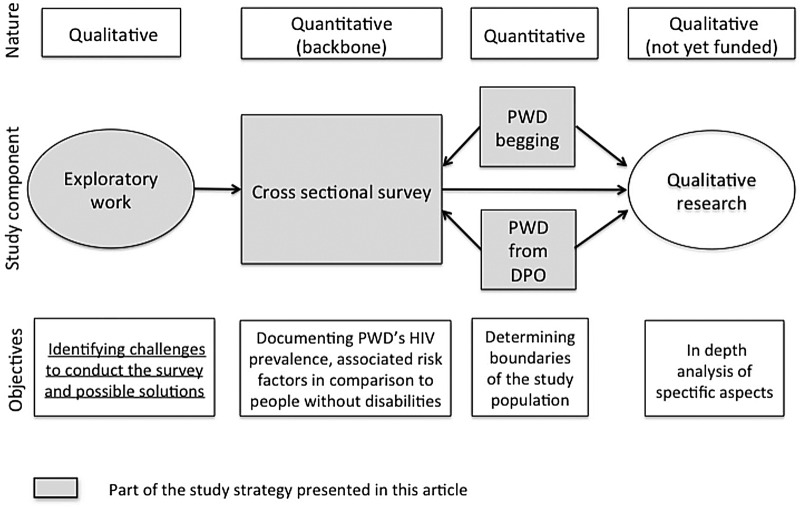
Study strategy.

This research project aims to combine qualitative and quantitative data on people with disabilities (figure 1). The quantitative component, a cross-sectional survey currently conducted in Yaoundé (Cameroon), constitutes the backbone of the research. Additional data—qualitative and quantitative—will complement the backbone component, providing complementary insight on the research question. An initial exploratory qualitative survey has been implemented to inform the subsequent quantitative work. Other qualitative research will be conducted after the quantitative survey to explore in more detail some of the quantitative findings. The two substudies on people with disabilities begging in the street and from disabled people's organisations use an exploratory quantitative design (see below).

Since the second qualitative research will be defined only after the analysis of the quantitative data, this article focuses on the cross-sectional component of the study, along with the exploratory work, and the two substudies.

Recruitment started in October 2014 and the estimated date of the last participants’ recruitment is November 2015.

### Backbone cross-sectional component

#### Washington group questionnaire (screening instrument)

To overcome the practical and conceptual difficulties in measuring disability, a group of experts set up by the UN Statistical Commission has proposed an operational tool for the identification of people with disabilities in surveys with good accuracy and reproducibility from one setting to another.^26^ ^47^ ^48^ This tool based on the International Classification of Functioning, Disability and Health (ICF) framework, includes a small number of questions covering six functional domains or basic actions: seeing, hearing, walking, cognition, self-care and communication. Each question asks the respondent to rate on a four-point scale how much difficulty he/she has experienced in the domain (see online supplementary appendix A). The Washington Group questionnaire is available in various forms; a short set questionnaire includes six questions and is recommended for use in national survey because of its simplicity. Additional questions are available from the extended set to supplement those from the short set and provide more detail on functional limitations.

##### Study population

All people aged 15–49 years with severe difficulties in at least one domain or some difficulties in at least two domains of the Washington Group questionnaire are considered as living with disabilities and therefore are eligible for the study.^47^ ^48^ For each person with a disability included in the study, a control of similar age group (within a range of 5 years) and sex, living in the same enumeration areas and without functional limitation criteria is also recruited.

##### Sampling methods and disability screening

The sampling procedure consists of two phases. In the first phase, 177 enumeration areas in Yaoundé were drawn from the national sampling frame provided by the Central Bureau of the Census and Population Studies. Each drawn enumeration area is enumerated again in an exhaustive way to update the data. In the second phase, up to 200 households are randomly selected in each of the enumeration areas. All members of the selected households aged 15 years and above are then screened for disability using eight questions from the Washington Group disability questionnaire (six questions from the short set and two additional questions from the extended set, see online supplementary appendix A).^47^ ^48^ The two additional questions from the extended set were included in the screening tool to better capture people with intellectual and/or mental disabilities.

##### Sample size

Sample size was computed to detect with a power of 80% and alpha risk at 5% a prevalence ratio >1.7 under the assumption that HIV prevalence in the controls is 6% in Yaoundé and that 10% of the participants may refuse the HIV test. A total of 850 persons with disabilities and 850 controls will be recruited. With this sample size, it is expected that each subgroup defined by impairments (hearing, visual, mobility, intellectual/mental) will include at least 100 participants. This is based on the assumption that the smallest subgroup will constitute at least 12% of the study population as it has been observed in other studies.^49^ ^50^

##### Outcomes

*HIV prevalence*: The primary outcomes are the prevalence of HIV among people with disabilities and the prevalence ratio compared to the control group. Prevalence and prevalence ratio will also be computed for subgroups defined by the following impairments: (1) hearing (2) visual (3) mobility (4) intellectual/mental.

*Knowledge, attitude and practices (KAP):* Knowledge, attitudes and practices on HIV and AIDS, sexuality and reproductive health are assessed by questions derived from the ‘Illustrative questionnaire for interview-survey with young people’ designed by Cleland *et al*.^51^ In addition, main events of their sexual and reproductive life are recorded during the life-event interview.

*Disability*: Given its complexity, two dimensions of disability, activity limitation and social participation restriction, are examined. The extended set of the Washington Group disability questionnaire is used to assess activity limitation.^48^ Although social participation is central to the definition of disability, there are many challenges in measuring social participation as defined in the ICF framework.^52–55^ Much of the issues result from the lack of a consensual definition of the social participation construct.^55^ In this study, difficulties in participation in community/family events and in the decision-making process are rated on a four-point scale using questions based on the work by SINTEF^56^ and Handicap International.^57^

*Environmental factors*: Questions derived from SINTEF's work based on the validated Craig Hospital Inventory of Environmental Factors (CHIEF) Short-Form are used to assess the effect of environmental factors on functioning and social participation.^58^ ^59^ Social support is assessed for all participants using questions adapted from the social network index.^60^

*Life-course trajectories:* Detailed information on the participants’ environment, history of employment, resources, relationships and reproductive health is collected during the life-event interview (see online supplementary annex B). It provides important information on the ‘life-situation’ aspects of social participation as mentioned in the ICF.^33^ It will be used to compare life-course trajectories between people with and without disabilities as well as people with disabilities across different types of impairments and their potential association with HIV status and adverse SRH outcomes.^61^

*Health services utilisation*: A short set of questions is used to determine the nature of SRH services used by the study respondents. Satisfaction with the last service used is also assessed. This topic will be investigated in more depth during the forthcoming qualitative component of the survey.

##### Interviews and methods

Face-to-face interviews are organised with eligible participants identified from the screening stage after informed consent is granted. These interviews are conducted in a confidential environment in the participants’ homes or in a specific place set by study interviewers specifically trained for this purpose. Eligibility of the participant is first confirmed with the Washington Group questions: participants eligible as people with disability are asked additional questions from the extended Washington Group set (17 questions) to have more details on their functional limitation while controls are asked the same eight questions from the Washington Group questionnaire as part of the quality control.

After eligibility is confirmed, the full interview is proposed to the person. It includes a life-event interview on participants’ history of social participation, employment, resources, sexual partnership and fertility using the life-grid method.^43^ ^62^ The life-grid is made of two A3 sheets divided into several columns (see online supplementary appendix B). The vertical axis represents the time; the first column gives time in years from birth to current year, the second column gives the age from 0 to current age and the third column gives the time elapsed. The other columns are used to report events according to their nature (people with whom the participant lived, main occupations/activities, resources, quality of life, sexual relationships, periods of transactional sex or sexual violence, pregnancies, children and disability onset). In addition, the occurrence of ‘important’ events is recorded as well as any information provided by the participants on the meaning of the events that occurred during his/her life. The life-grid helps to recall and structure the history of life events into chronological categories by cross-referencing the events. The grid is continually shown to the study participants as biographical information is recorded by the interviewer. The interview also includes closed questions on the participants’ characteristics, activity limitations, knowledge, attitudes and practices on HIV and SRH, environmental factors, social support and participation.

Several questions probing exposure to physical and/or sexual violence are asked at different moments of the interview. These questions are intended only to screen for violence and not to investigate the nature or circumstances of these events. However, the qualitative component of this study will aim to give a better insight into the exposure to violence. Where needed (eg, with deaf people or with people with intellectual disability), pictograms and other communication tools (like dolls, drawings and images) are used to facilitate communication between study interviewers and respondents. All interviewers received intensive training on study procedures, interview methods, as well as on disability and methods of communications with people with disabilities. Two interviewers are proficient in American and French sign languages and two have physical disability.

##### HIV testing

Each participant is offered voluntary HIV counselling and testing. It is carried out at the participant's home, or at any other place where there is assurance of confidentiality if the participant prefers. The counselling is led according to international guidelines. Communications strategies are adapted to each type of disability through the use of tools such as pictograms, dolls, drawings and wooden penis when relevant. Two rapid blood tests are used following national and international guidelines.^63^ ^64^ HIV infection is initially screened using the sensitive rapid blood test Parallel Determine (Abbott, Japan) and further confirmed using the INSTI HIV-1/HIV-2 (bioLytical TM). An HIV ELISA antibody laboratory test will be performed in case of discordant results between the two rapid tests and for 10% of all tests as part of the quality control. All specimens are identified only through the anonymous study number to ensure confidentiality. For participants who refused HIV testing, a phone number is given for them to contact if they change their mind.

##### Statistical analysis

A weighted estimator will be used for prevalence and proportion estimates.^36^ ^65^ CIs will be adjusted for the sampling design using the bootstrap method and the Rao and Scott corrected Pearson test will be used to compare proportions.

Potential risk factors for HIV will be identified using multilevel regression. Three categories of variables will be considered: individual characteristics, environmental characteristics and life-course characteristics. Multistate models will be used to analyse sequences of events and life transitions experienced. Social sequences from the life-course grid will be analysed using optimal matching algorithms.^66^ First, an overall analysis will be performed without accounting for the presence of a disability. This will provide a number of typical life trajectories. In the second step, the association between specific life trajectories and the presence of specific impairments and/or social participation restrictions will be assessed as well as the association between typical life trajectories and HIV vulnerability indicators. Univariate and multivariate logistic regression models will be used for these analyses.

### Exploratory work: results and study adaptation

Before starting the cross-sectional component, a mapping of the various organisations for people with disabilities was completed using a snowball method until saturation of the information. Additionally, a qualitative survey was implemented to assess the acceptability of the study procedures and determine possible barriers to reach people with disabilities. Using a purposive sampling method, a total of 10 semistructured individual interviews and 3 semistructured focus groups (each with 6 persons) were conducted. Focus groups were conducted separately with males and females. Interviews were carried out with people having visual, hearing and physical impairments, relatives of people with intellectual impairment and professionals working with people with disabilities. All interviews were audio recorded and transcribed. Transcripts were manually coded and analysed using thematic content analysis. All the interviewed persons with disabilities fulfilled the inclusion criteria of the HandiVIH study (age and activity limitations according to the Washington Group questionnaire). The exploratory interviews revealed five main themes that need to be considered in the implementation of the study. (1) Most of the key informants mentioned the frequent exposure of women with disabilities to physical and sexual violence. This was accounted for in the study implementation through the identification of referral organisations for victims of violence and specific training of the interviewers on that topic. (2) All key informants insisted on the importance of developing a trusting relationship during the interview with the person with a disability. The inclusion of people with disabilities in the interviewers’ team was a successful way to do that. The life-event interview also contributed to such a relationship. Besides, prior to launching the study, sensitisation sessions have been conducted in each enumeration area through media, reeves and traditional chiefs to increase the trust of families in the study. (3) Some key informants highlighted the fact that interviewers might need to be accompanied by another familiar person with a disability to gain the trust of some people with disabilities, a strategy that is used when necessary, and more often with persons living on the street. (4) Many key informants also stressed that people with intellectual disabilities may be hidden to the research team because of feelings of shame commonly found in their community. To overcome this issue, questions about the composition of the household are asked several times in different ways and at different periods of the enumeration and screening phases. In addition, interviewers have been trained and supported in developing interpersonal skills to get the trust of the first respondent of the household. (5) To finish, key informants explained that in Yaounde, since there is only one institution that can accommodate people with disabilities, most of them live in households. They also hold the view that most of the people with disabilities begging in the street belong to a household and only few of them actually sleep on the street.

The interview tools for the different phases of the survey were field-tested and adapted. Standardised reformulation of the Washington Group questions was prepared using the user and cognitive testing guidelines available from the website and from one of the co-authors (DM).

### Substudies: towards a better understanding of the disabled population throughout the spectrum

The backbone cross-sectional component might fail to include people with disabilities living on the streets. To address this concern, a substudy has been added that recruits beggars with disabilities through a purposive sampling strategy. After consent is granted, the same questionnaire and HIV counselling/testing as for the cross-sectional component participants are proposed to beggars with disabilities. In addition, information on their living conditions and on their social network is collected during the interview.

While most available studies recruit their study populations from DPOs’ networks, it has been pointed out that the characteristics of these populations may differ from those of the general population of people with disabilities who do not adhere to any groups or organisations. In the second substudy, members of the DPO mapped during the exploratory work are randomly recruited and offered to participate in the interviewing and to get an HIV testing/counselling after informed consent. Using the same statistical method as for the cross-sectional components, the characteristics of this subgroup will be compared with those of the participants in the main study to better understand to what extent data obtained from DPO members may reflect the situation of the overall population of people with disabilities.

For these two substudies, no formal sample size calculation has been performed because of their exploratory nature. However, a sample size of at least 50 participants in each sub-study was targeted in order to provide a power of 80% and to obtain results with a precision of at least 15%.

### Ethical issues

The final protocol has been approved by the ‘Comité d'Ethique pour la Recherche en Santé Humaine’ in Cameroon, and ‘Comité Consultatif de Déontologie et d'Ethique’ from the Institut de Recherche pour le Développement (IRD). Special attention has been paid to giving adapted information to persons with intellectual disabilities (using simple language and pictures) and involving them in the consenting process. Emphasis was also given to ensure confidentiality and privacy during the interview and HIV testing. Since some questions explore sensitive topics that may cause emotional distress, interviewers were trained to first provide psychological aid^67^ and to refer people requesting assistance to available services in Yaounde. All participants diagnosed with HIV infection received information about antiretroviral treatment and places where they could receive care. In addition, the study nurse proposes to those who are disabled to escort them at their first medical visit in order to decrease possible barriers.

## Discussion

To the best of our knowledge, this study will be one of the most comprehensive surveys on HIV and SRH to be conducted among people with disabilities in Sub-Saharan Africa. It will use a rigorous methodology to provide quantitative data on the burden of HIV among people with disabilities as well as information on their life situation.

In the cross-sectional component, the study attempts to overcome several methodological difficulties in identifying persons with disabilities that deserve attention. First, a relatively complex sampling design has been chosen to overcome the problem of identifying people with disabilities in the absence of a sampling frame, using a two-phase cluster random sampling with screening of disability at phase one. The rationale for this design is to use a first simple screening tool (here the Washington Group short set+2 questions) to reduce the population in which the more complex ‘diagnosis’ tool (here the Washington Group extended set) is used. Compared to snowball sampling that has been used in most available surveys, this sampling design decreases significantly the risk of selection bias. Available studies from Sub-Saharan Africa on the health of people with disabilities tend to rely on populations of people with disabilities identified from DPO or institutions (eg, schools) rather than from the general population. Data collected so far in the HandiVIH study confirm that the majority of people with disabilities recruited do not report connection to any known DPO, which suggests that populations of people with disabilities identified from DPOs may differ from the general population of people with disabilities. A trade-off had to be made between simplicity, ease, and speed of use of the screening tool and its sensitivity in order not to miss cases. In this study, it was decided to include two additional questions to the Washington Group short set to improve its sensitivity to detect intellectual or mental impairment because it was assumed that people with these impairments may be at an increased risk of sexual abuse and violence. The main potential limitation of this two-phase design is the inability of the screening tool (Washington Group questionnaire) to correctly partition the population into two groups defined by the disability status.

Various tools have been used to identify people with disabilities in the surveys and comprehensive discussions of their limitations have already been published.^13^ ^26^ ^34^ ^68^ In this study, the two important features sought for the tool used to measure disability were its coherence with the ICF disability model and its ability to provide valid and reliable data that could be compared with similar data from other countries.

Currently, the Washington Group questionnaire is increasingly being used, recommended for use, and the most validated questionnaire for screening disability in adults.^69^ Although, to the best of our knowledge, it has not been used for HIV survey among persons with disabilities to date, the WG questionnaire has been used in other surveys on health,^70^ ^71^ and is likely to be included in future Demographic Health Surveys (D Mont personal communication). Nevertheless, this tool is based on the self-report of functional limitations and may not identify clinical impairments if the respondent does not consider his/her impairment as a limitation (so-called response shift in the field of quality of life assessment^72^ ^73^). Two recent studies conducted in Cameroon and India have found that up to 46% of the people with disability identified through clinical impairment screening methods were missed by the Washington Group questionnaire.^74^ ^75^ Another important issue is the determination of a cut-off in the level of difficulties that will differentiate people who are potentially with disabilities from those without. In this study, using the experience from two other surveys,^56^ ^71^ it was decided that anyone with a major difficulty in at least one domain or some difficulties in at least two domains would be considered eligible as people with disabilities. To detect possible disability measurement errors done with the eight questions of the Washington Group questionnaire at the screening phase, disability eligibility is confirmed with the Washington Group extended set before enrolment in the second phase of the study. Lastly, it should be emphasised that the identification of functional limitations is only a first step to the identification of disability and needs to be complemented with additional questions on social participation and environment as well as impairment screening.^27^ ^33^

An important feature of this research is the combination of different approaches to collect information on the study population. The quantitative cross-sectional survey constitutes the backbone of the project. However, since the population of people with disabilities is heterogeneous, complex and understudied, this quantitative component is completed with substudies having an emergent design. This heuristic approach is expected to give a more flexible and comprehensive understanding of the studied population while maintaining scientific rigour and quality. A first sub-study was defined to get a better understanding of the situation of the people with disabilities begging who may not have been included in the cross-sectional study. The second substudy, focusing on people with disabilities who are members of a DPO, is expected to provide more insight into possible limitations of epidemiological results based only on membership of DPO. These data are also expected to help to better adapt interventions to specific groups of people with disabilities (eg, beggars, members of DPO). As results accumulate, other qualitative researches connected to the project may be defined.

Another major challenge of this study is to get a better understanding of the mechanisms explaining the vulnerability of people with disabilities to HIV. It should be emphasised that the cross-sectional design of the study precludes any firm causal conclusion as could be done in a prospective cohort. However, the life-course approach used in the study may provide us with important information. The strength of the life-course approach lies in its ability to provide a comprehensive insight into the context, connecting sexual risk behaviour to other social factors and looking at events with an adequate time perspective.^76^ The life course perspective is particularly important in the context of people with disabilities who experience disadvantages that accumulate throughout the life.^77^ Besides, the life-grid method for collecting biographical information has been shown to improve the recall and thereby the accuracy of collected data.^44^ Retrospective data collection might not be as accurate as in prospective studies, although it was shown to provide information with good validity and reliability for some types of events.^78–80^ Moreover, the subjective assessment of events and life circumstances during the life-event interview is important information that may contribute to a better understanding of the life situation of people with disabilities and its relation to HIV risk. Interestingly, the life-event method gives more attention to the respondents’ perspective. Owing to this opportunity offered to respondents to account for their lives, the life-event method creates an environment conducive for sharing intimacy, which contributes to improving the quality and wealth of the data. It also gives the possibility to combine individual and social data. Although possible, the use of the life-grid method with people with intellectual disabilities is challenging. In our experience, a third person close to the respondent has to help in answering to the environmental activities and resource modules. Nevertheless, for most of this specific disabled population, the training of the interviewers as well as the use of the communication tools (pictograms, dolls) has allowed the collection of intimate data without the help of a third person, preventing the proxy bias in important topics such as sexuality and intimate relationships.

In conclusion, this study aims at providing comprehensive information on the vulnerability of people with disabilities to HIV and in their SRH. To address the multiple challenge of providing comprehensive and valid data on the vulnerability of people with disabilities to HIV and SRH adverse outcomes, an innovative approach is used, which needs to be shared and discussed with other stakeholders. The key aspects of this approach are (1) adoption of a disability-inclusive approach as much as possible; (2) use of an exploratory phase to adapt processes and tools to the specificities of the target population; (3) use of a random population-based sampling and of (4) standardised ICF-oriented questions to select people with disabilities in the study; (5) adaptation of the various tools to participants’ needs and (6) adoption of a life-course approach to better understand HIV and SRH vulnerability specifically related to disability. The findings of this research are expected to bridge important gaps in the knowledge and to inform decision-makers of the development of more accessible and appropriate HIV/SRH-related interventions for people with disabilities. They are also expected to stimulate additional research either to confirm these findings in other African settings or to broaden some aspects of the results.
